# Supplementary material for: Development of a Novel Anti-CD44 Variant 4 Monoclonal Antibody C44Mab-108 for Immunohistochemistry
Source: Curr Issues Mol Biol. 2023 Feb 25;45(3):1875–88. doi: 10.3390/cimb45030121 (PMC10047040; doi:10.3390/cimb45030121)
Supplement: Supplementary file 1 [file cimb-45-00121-s001.zip › rev supplemental Figure S1-S3 .pdf]

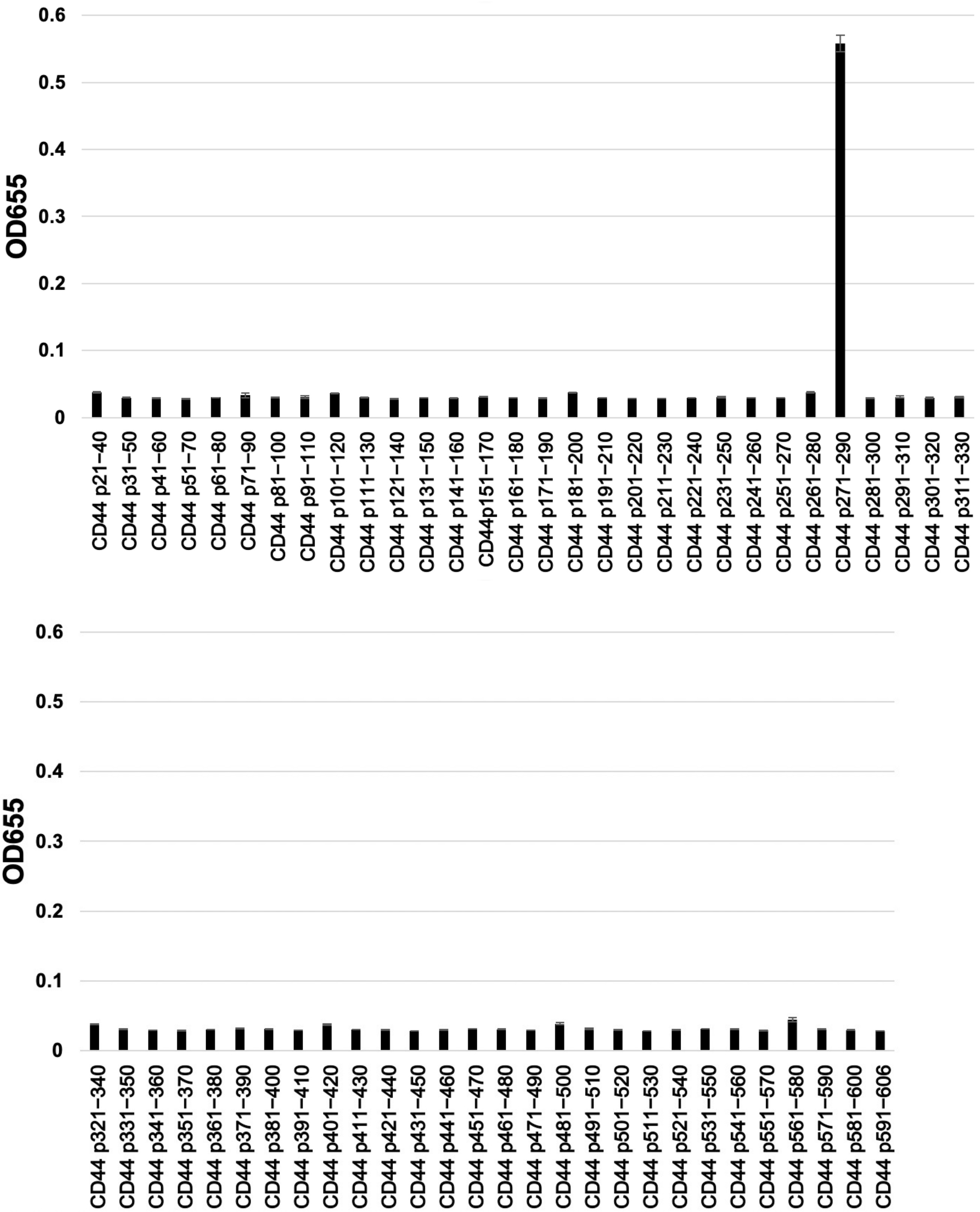

Supplementary Figure S1 Conformation of C_{44}Mab-108 epitope by ELISA using peptides which cover the CD44v3-10 extracellular domain. Fifty-eight synthesized peptides [17] were immobilized on immunoplates. The plates were incubated with C_{44}Mab-108 (1 μg/mL), followed by incubation with peroxidase-conjugated anti-mouse immunoglobulins. Optical density was measured at 655 nm. Values are presented as the mean ± SD. ELISA, enzyme-linked immunosorbent assay.

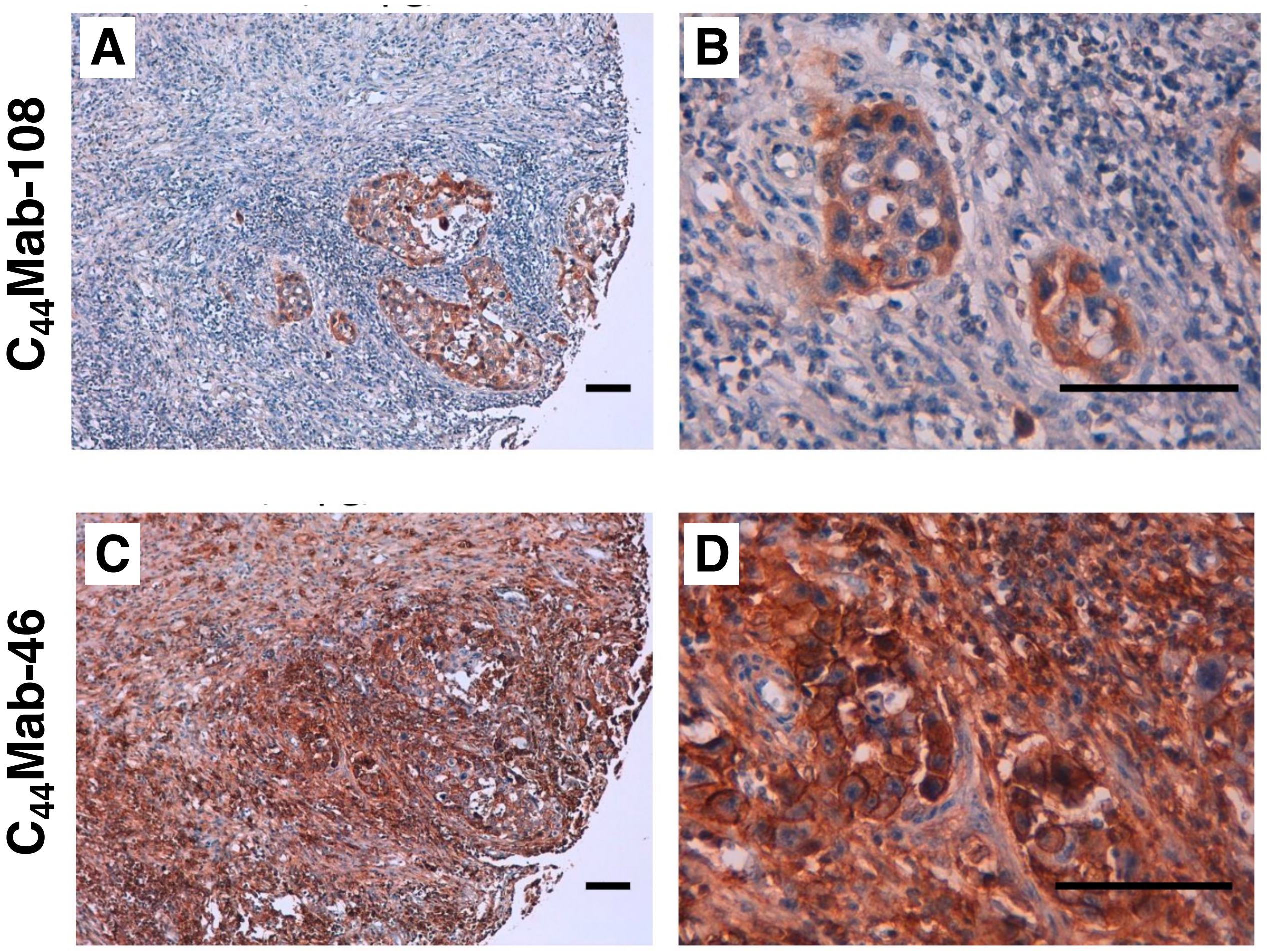

Supplementary Figure S2 Immunohistochemical analysis using C_{44}Mab-108 and C_{44}Mab-46 against esophageal squamous cell carcinoma tissues. After antigen retrieval, the esophageal tissue microarray (BC02011, US Biomax Inc.) were incubated with 10 μg/mL of C_{44}Mab-108 (A and B) and 1 μg/mL of C_{44}Mab-46 (C and D), followed by treatment with the Envision+ kit. The color was developed using 3,3′-diaminobenzidine tetrahydrochloride (DAB), and the sections were counterstained with hematoxylin. Scale bar = 100 μm. As with OSCC (Fig. 4), C_{44}Mab-108 stained stromal invaded tumors and could clearly distinguish tumor cells from stromal tissues (A and B). In contrast, C_{44}Mab-46 stained the both (C and D).

```
Human  DHTKQNQDWTQWNPSHSNP
Mouse  ARTEDNQDWTQWKPNHSNP
Rat    AHTKQNQERTQWNPIHSNP
Cham   DHTKQNQDWIQWKPSHSNP
```

Supplementary Figure S3 An alignment of human, mouse, rat, and Chinese hamster (Cham) CD44 vriant 4 sequences. In our preliminary epitope mapping analysis, alanine-substitution of each amino acid (allows) in human v4 region reduced the reactivity of C_{44}Mab-108 (manuscript preparation). Since amino acid sequence of the region is identical between human and mouse (underlined), C_{44}Mab-108 could recognize both human and mouse CD44v4.
