# Supplementary material for: Development of a Novel Anti-CD44 Variant 4 Monoclonal Antibody C44Mab-108 for Immunohistochemistry
Source: Curr Issues Mol Biol. 2023 Feb 25;45(3):1875–88. doi: 10.3390/cimb45030121 (PMC10047040; doi:10.3390/cimb45030121)
Supplement: Supplementary file 1 [file cimb-45-00121-s001.zip › Supplementary Table S1.pdf]

Supplementary Table S1. Determination of C_{44}Mab-108 isotype by ELISA.

	C_{44}Mab-108
Ig	+
IgG_1	+
IgG_{2a}	-
IgG_{2b}	-
IgG_3	-
IgM	-
kappa	+
lambda	-

+, OD655≧0.2; −, OD655<0.05

Ig, immunoglobulin

ELISA, enzyme-linked immunosorbent assay
